# Development of a rapid and inexpensive method to reveal natural antisense transcripts

**DOI:** 10.1186/1746-4811-8-37

**Published:** 2012-09-12

**Authors:** Silvio Collani, Gianni Barcaccia

**Affiliations:** 1 Laboratory of Plant Genetics and Genomics, DAFNAE – University of Padova, Campus of Agripolis, Viale dell’Università 16, 35020 Legnaro, PD, Italy

**Keywords:** Natural antisense transcripts (NATs), Plant NATs, Olive

## Abstract

**Background:**

Natural antisense transcripts (NATs) are a group of RNAs encoded within a cell that have transcript complementarity to other RNA transcripts. NATs have been identified in multiple eukaryotes, including humans, mice, yeast and several plants, and are known to play crucial roles in gene regulation and modification via RNA interference, alternative splicing and genomic imprinting. NATs are also involved in several human diseases.

**Results:**

We describe a novel method to detect the occurrence of target NATs in specific plant tissues. This method differs from the others currently used in molecular biology laboratories for a number of reasons, particularly the simplicity and versatility of application, low cost and lower material requirement. We demonstrate that NATs can be detected by using diluted cDNA, avoiding the need for a large amount of RNA, thus differing from basic techniques, such as Northern blot hybridisation and reverse-transcription PCR amplification. Furthermore, our method also allows the precise detection of long NATs and their cloning into plasmid vectors for downstream applications. We also reported the first case of a tissue-specific NAT occurring in *Oleaceae* family and, the antisense orientation of this transcript, allows the splicing of two introns otherwise impossible in the sense orientation.

**Conclusions:**

This method is the first that combines the polymerisation and cleavage activity of DNA polymerase and exonuclease enzymes, respectively, to discover NATs in living organisms. It may simplify the discovery of NATs in plants providing a new strategy for an easy identification and characterization of this group of RNA molecules. Furthermore, since NATs are found in multiple eukaryotes, our method can be easily applied to a wide range of organisms, including human, mice and yeast.

## Background

Natural antisense transcripts (NATs) are RNAs with sequences that have complementarity to other endogenous RNAs [1]. NATs are classified into two main categories: *cis*-NATs, transcribed in *cis* from opposing DNA strands at the same genomic locus, and *trans*-NATs, transcribed in *trans* at separate loci [1]. The *cis*-NATs, which are transcribed from the reverse-complementary strand of an annotated gene and, hence, are fully or partially overlapping with their respective mRNAs, are also known as antisense RNAs (asRNAs) [2]. Several methods were developed to predict asRNAs in different species, and it was estimated that asRNAs occur for a proportion of annotated genes equal to 22-26% of the human genome [3-5], 15-29% of the mouse genome [5-7], and approximately 9% of the *Arabidopsis thaliana* genome [8,9]. Databases that include thousands of predicted NATs for both the animal and plant kingdoms were also developed and are now available on-line [5,10].

In recent years, NATs were found to be implicated in a wide range of aspects of gene expression in eukaryotes, including genomic imprinting, RNA interference, translational regulation, alternative splicing, X-inactivation and RNA editing [1,11-15]. It is also known that NATs may be involved in several human diseases and in several responses to stress in plants [14,16].

In plants, several NATs are predicted and annotated [10]: they are usually quite small in size and have a nuclear origin, but long and chloroplast asRNAs are also known [2].

In the last decade, the massive application of NGS technologies allowed the implementation of the large repertoires of genomic and transcribed sequence data for the *in silico* prediction of NATs [5,17-23]. Nevertheless, an *in vitro* validation of their presence in specific cell populations and the timing of expression are crucial to determine the role played by a given NAT. To date, the detection of NATs within tissues or organs is achieved by reverse transcription PCR-mediated amplification (RT-PCR) and Northern blot hybridisation techniques, which are based on the use of a large amount of total RNA and require RNase-free reagents and disposables to avoid degradation of the RNA.

Moreover, Northern blot hybridisations are time consuming and require labelled probes, and certain RNAs produce secondary structures during the reverse transcription step of RT-PCR that can act as primers for retro-transcriptase, hence determining primer-independent cDNA synthesis. It has been shown that retro-transcriptases that also have RNase H activity [specially designed for reverse transcription with different amount of RNA and any additional RNase H digestion step, such as Omniscript® Reverse Transcription (Qiagen)] can reduce the amount of primer-independent cDNA synthesis [24], but the cost of this enzyme remains relatively high, and the method needs to be optimised for each case. Furthermore, the presence of RNase H activity during first-strand synthesis may increase the degradation of the template mRNA, resulting in decreased full-length and first-strand cDNA [see technical note reported in protocols of the most common reverse-transcriptase enzymes, such as SuperScript® III (Invitrogen)]. Our method overcomes these problems as it is based on the use of a cDNA template. However, it is also possible that single-strand cDNA might produce secondary structures and, thus, prime DNA-dependent DNA-polymerase, producing primer-independent DNA synthesis. Using tagged primers that contain a known sequence at the 5’ end and specific primers designed on the known sequence (TAG) during the next amplification is sufficient to overcome this technical issue [25].

Here, we report a simple method to detect NATs within a population of cDNAs that avoids the direct use of RNA, as it is based on diluted cDNA templates (up to 1:25) retro-transcribed from small amounts of total RNA (up to 100 ng). Theoretically, if a pair of primers is able to amplify a gene in a diluted cDNA sample, then the method can be efficiently applied for the discovery of NATs. It is worth noting that, during RNA retro-transcription using gene-specific primers (GSPs), some GSPs fail to prime cDNA synthesis, even though they work well in PCR using DNA templates (see technical note reported in protocols of the most common reverse-transcriptase enzymes). Our method is more reliable because it is based on the same cDNA template as PCR.

**Figure 1:**
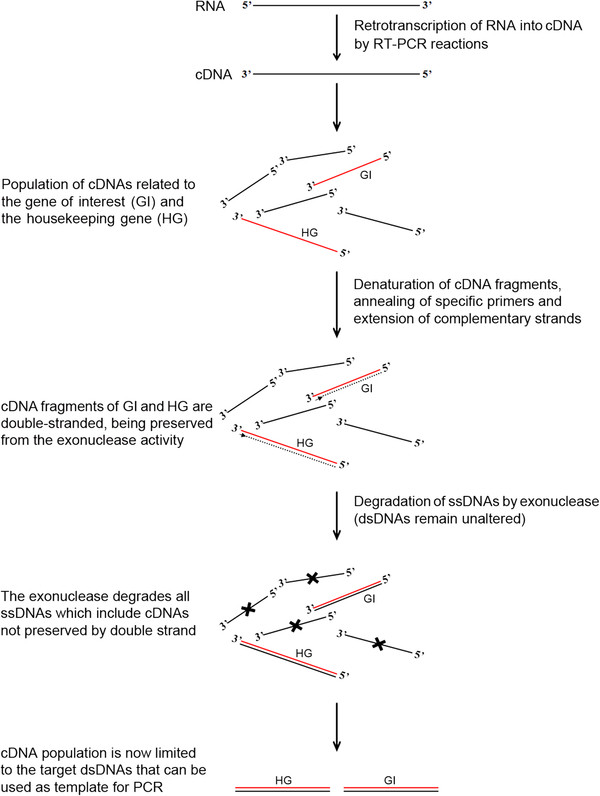
**Schematic representation of the method used to detect NATs within a cDNA population.** Total RNA is retro-transcribed into cDNA, which was then used as the template to produce complementary strands of the transcripts that showed homology with the primers added to the reaction. DNA-dependent DNA polymerase synthesised the complementary strands of the target genes, which are thus preserved from exonuclease degradation. All of the ssDNA will be degraded, leaving those transcripts with complementary strands intact. PCRs were then performed to detect whether the target gene was present. Red line: cDNA that will be protected by the synthesis of its complementary strand. HG: reference gene used as the positive internal control. GI: gene of interest, the gene for which the present of antisense was examined.

The method consists of four main steps, as follows: i) the retro-transcription of RNA using oligo(dT) or random primers to produce first-strand cDNA of the 3’-poly(A) tail RNAs and total RNAs, respectively; ii) the synthesis of the second strand of any target cDNA to obtain double-stranded complementary DNA; iii) the cleavage of all single-stranded DNA molecules using an exonuclease that specifically recognises them, releasing deoxyribonucleoside 5’-monophosphates in a stepwise manner; and iv) the PCR-mediated amplification of the target genes using specific primers (Figure 1).

Our method was developed and tested using an anther-specific NAT found in olive (*Olea europaea* L.) that is related to *SLG* of *Brassica*, *OeSLG*, a gene encoding a protein that functions as an enhancer of the self-incompatible response in some *Brassica* lines, even though the presence of the protein is not crucial for the rejection of self-pollen. Our overall results demonstrated the usefulness of the method to reveal natural antisense transcripts, and its distinctive features are critically discussed.

## Results

### Detection of tissue specific transcripts

**Figure 2:**
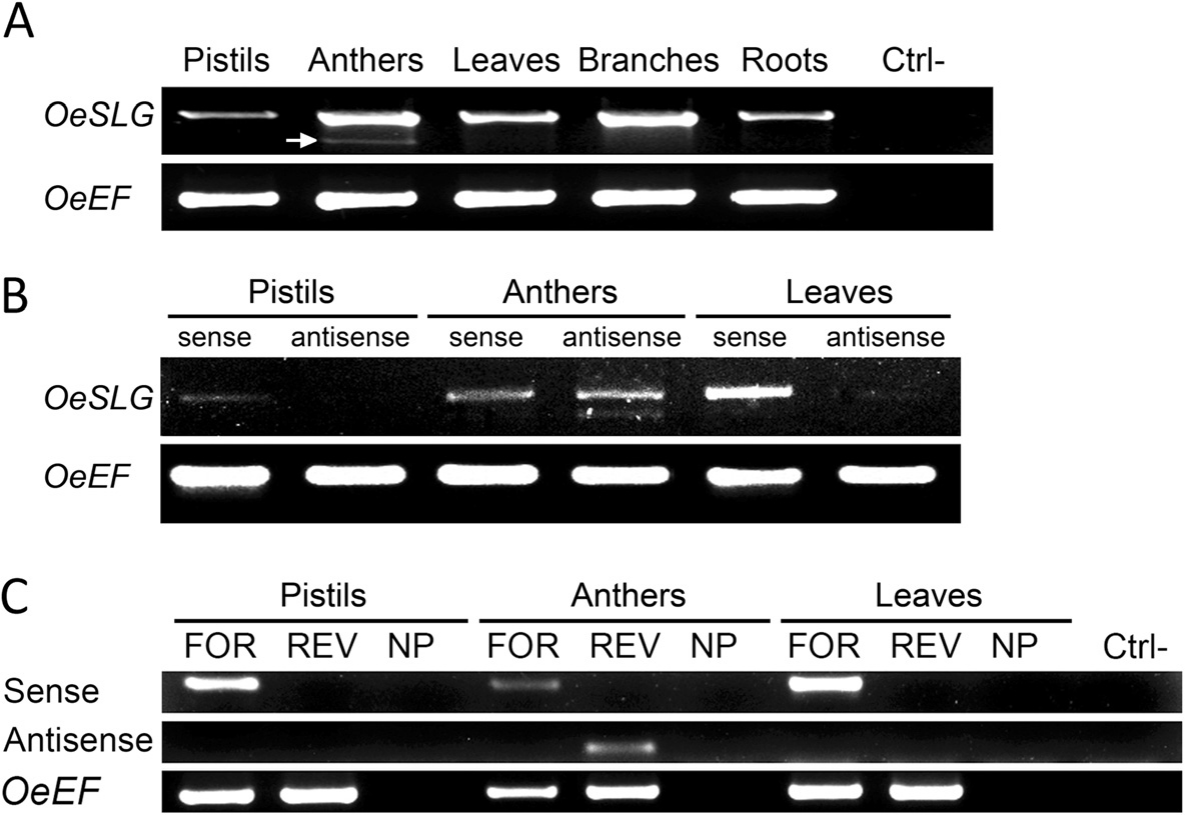
**PCR amplifications on cDNAs to reveal anther-specific NATs in olive. A**. RT-PCR analysis in different tissues using primers for the start and stop codons, OeSLG_full-length_FOR and OeSLG_full-length_REV, respectively, to amplify the target gene, *OeSLG *, and using primers OeEF_FOR and OeEF_REV to amplify the housekeeping gene, *OeEF*. Ctrl-: negative control. A lower band (indicated by an arrow) is present only in anthers. **B**. Final PCR amplifications of the *OeSLG *gene were performed using primer pairs suitable to detect either the sense transcripts or the antisense transcripts. The housekeeping gene *OeEF *was amplified using specific primers. Sense transcripts are detected in all of the tissues, whereas the antisense transcripts are found only in anthers and are present by two different forms, corresponding to the medium and short forms, as expected. **C**. Final PCR amplifications were performed after the synthesis of the complementary strands as follows: FOR, OeSLG_full-length_5’-tag_FOR; REV, OeSLG_full-length_5’-tag_REV; NP, no primers were added to reactions. PCR analysis was performed using the following combination of primers: sense, primer_TAG and OeSLG_internal_5’_REV; antisense, primer_TAG and OeSLG_internal_3’_FOR; *OeEF *, primer_TAG and OeEF_REV; Ctrl-, negative control. Sense transcripts are detected in all tissues, whereas antisense transcripts are found only in the anthers. The negative control showed that the possible complementary strands synthesised in a primer-independent manner were not amplified. The antisense transcripts resolved as single band because the internal primers used were external to the introns and they were not able to discriminate between the medium and short forms.

A gene, *OeSLG* (*Olea europaea* SLG), related to *SLG* of *Brassica*, which encodes an enhancer of the self-incompatibility response in some *Brassica* lines, though its presence is not essential to reject self-pollen, was previously isolated in our lab [26]. RT-PCRs performed using RNA from pistils, anthers, leaves, branches and roots revealed that two transcripts were present in the anthers, whereas only one was present in the other tissues (Figure 2, panel A). 

The amplicons were cloned and sequenced. Intriguingly, three forms of *OeSLG* were isolated from anther tissue: one sense *OeSLG*, as in the other tissues, and two forms differing from the sense *OeSLG* by the lack of one or two internal regions. In detail, the three transcripts consisted of one long form (1,269 bp), equivalent to that expected for *OeSLG*, one intermediate form (1,181 bp), lacking an 88 nt region, and one short form (780 bp), which was characterised by the lack of the same 88 nt region and an additional 401 nt region (Additional file 1). When we compared the antisense sequences of the intermediate and the short forms to that expected from the *OeSLG* coding sequence, we found that both missing regions were flanked by the splice donor (GU) and the splice acceptor (AG) sequence sites. We speculated that these regions were introns excised from the antisense sequences under the control of an anther-specific promoter. To demonstrate our hypothesis, we tested our new method to reveal the putative NATs.

### Detection of NAT using different tissues

The method was applied to three different tissues, pistils, anthers and leaves, using one 5’-tagged forward primer designed to anneal to the start codon of the *OeSLG* gene (OeSLG_full-length_5’-tag_FOR) to detect the sense transcripts and one 5’-tagged reverse primer designed to anneal to the stop codon of the *OeSLG* gene (OeSLG_full-length_5’-tag_REV) to detect the antisense transcripts. One 5’-tagged forward primer (OeEF_5’-tag_FOR) was also added to each reaction to detect the sense transcripts of the *OeEF* gene used as housekeeping gene. The final amplifications of the *OeSLG* gene were performed using one primer designed to anneal to the TAG (primer_TAG) and GSPs designed to anneal to the stop codon (OeSLG_full-length_REV) and start codon (OeSLG_full-length_FOR) to amplify the full-length sense and antisense transcripts, respectively. *OeEF* was amplified using the TAG primer and one internal reverse GSP (OeEF_REV). The results showed that the sense transcripts of *OeSLG* were present in the pistils, anthers and leaves, whereas the antisense transcripts of *OeSLG* were present only in the anthers (Figure 2, panel B).

As expected, two different antisense forms were detected in the anthers because the two different splicing variants were found to be specifically expressed in this organ: the intermediate (1,181 bp) and short (780 bp) forms. To validate the first result, we also tested internal primers for *OeSLG* and *OeEF*. As in the above experiments, the sense transcripts of the *OeSLG* gene were detected using the forward 5’-tagged primer (OeSLG_full-length_5’-tag_FOR) and its antisense transcripts using the reverse 5’-tagged primer (OeSLG_full-length_5’-tag_REV); the sense transcript of the *OeEF* gene was detected using the forward 5’-tagged primer OeEF_5’-tag_FOR. A further reaction was performed without adding primers to test the synthesis of the complementary strands in a primer-independent manner. The final amplifications were performed using the TAG primer in combination with the internal reverse GSP OeSLG_internal_5’_REV to detect the sense transcripts, the internal forward GSP OeSLG_internal_3’_FOR to detect the antisense transcripts, and the reverse GSP OeEF_REV (Figure 2, panel C). The results showed the expression of the sense *OeSLG* transcripts in the pistils, anthers and leaves and also confirmed the specificity of the antisense transcripts only in the anthers. The reactions performed without adding primers showed no signal, meaning that the amplicons obtained were reliable.

Our method proved to be suitable to demonstrate the presence of antisense transcripts specifically expressed in anthers, and this orientation allowed the splicing of introns otherwise not possible in the sense orientation due to the lack of the splice donor and acceptor sites.

### Interaction between sense and antisense OeSLG transcripts in anthers

**Figure 3:**
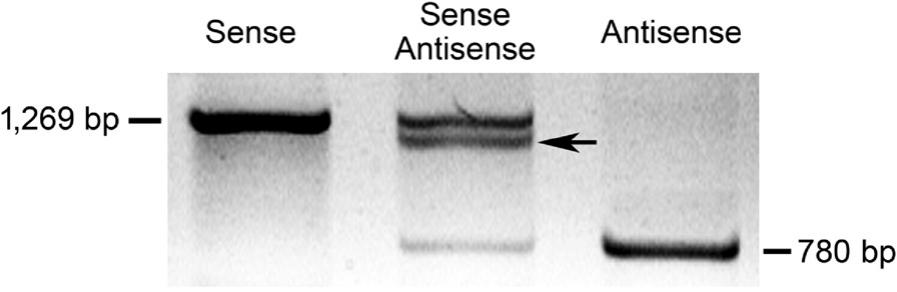
**Test of interaction between sense and antisense OeSLG transcripts in anthers. **The full-length long and short forms were cloned, amplified, precipitated, denatured, annealed and separated on an agarose gel in three combinations: sense/sense, sense/antisense and antisense/antisense. The sense/sense transcripts show a single band of 1,269 bp, the antisense/antisense transcripts show a single band of 780 bp, and the sense/antisense transcripts show three different bands, one higher, one lower and one of intermediate size, indicating that interactions between the sense and the antisense transcripts are possible.

To test the possible interaction between the sense and antisense *OeSLG* transcripts in the anthers, an *in vitro* annealing approach was performed, consisting of separating the full-length sense *OeSLG* (long form) and the shorter antisense *OeSLG* (short form) using agarose gel electrophoresis, with one well loaded with the combination of the sense and antisense forms. Theoretically, the sense *OeSLG* will produce a single band of 1,269 bp, the antisense *OeSLG* a single band of 780 bp (spliced form) and the mixed reaction will produce three bands, one of 1,269, one of 780 bp and another band characterised by an intermediate size between 780 bp and 1,269 bp because of the interaction between the entire sense and the spliced antisense transcripts (Figure 3).

The results showed the expected bands, meaning that interaction between the two forms, the entire sense and spliced antisense *OeSLG*, is possible.

### RT-PCR analysis with gene-specific primers

**Figure 4:**
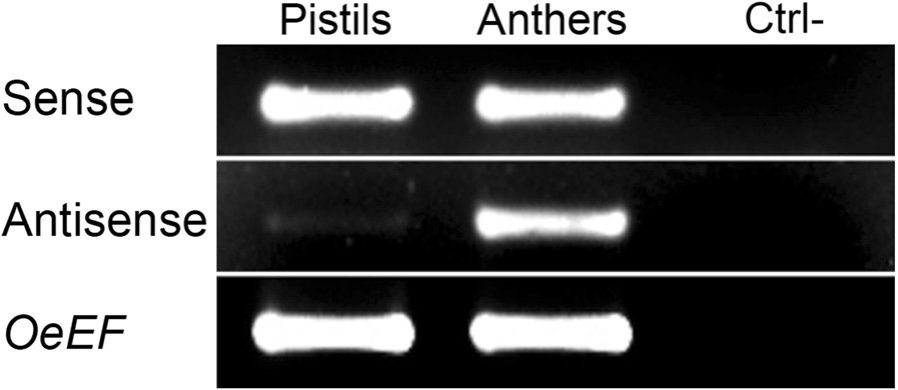
**Validation of the method through RT-PCR using gene-specific primers. **Sense: RT-PCR carried out using OeSLG_full-length_5’-tag_REV and subsequent amplification using primer_TAG and OeSLG_internal_FOR. Antisense: RT-PCR carried out using OeSLG_full-length_5’-tag_FOR and subsequent amplification using primer_TAG and OeSLG_internal_REV. The sense *OeSLG *was expressed in both pistils and anthers, whereas the antisense *OeSLG* was expressed almost exclusively in anthers. OeEF: Elongation Factor α1 used as positive internal control.

RT-PCRs were performed using gene-specific primers in order to further validate our method. Results showed that sense transcripts were present in both pistils and anthers, whereas the antisense transcripts were specifically expressed in anthers (Figure 4). This finding demonstrates the reliability of the method and proves that it is robust and can be applied as a rapid and inexpensive method to reveal natural antisense transcripts.

## Discussion

Our method provided several advantages compared to the methods normally used to detect NATs, such as Northern blot hybridisation and RT-PCR amplification. First, the present method avoids the use of large amounts of RNA and all of the problems associated with the use of RNA, such as the risk of RNA degradation and requirement of RNase-free conditions. It is also occasionally difficult to collect a large amount of RNA from some tissues or organs, therefore working with diluted cDNA allows for larger number of experiments using the same amount of RNA. Moreover, some fresh tissues are not available throughout the year, such as woody plants, which cannot be grown in greenhouses and that flower only once per year. Thus, the first-strand cDNA synthesised from limited amount of RNA can be diluted and used as a template, assuring that experiments can be conducted without waiting for the next flowering period.

It is known that during RNA retro-transcription using GSPs, a common method used to detect NATs in specific tissues or organs, some GSPs fail to prime cDNA synthesis, even though they work well in PCR using DNA templates (technical note reported in protocols of the most common reverse-transcriptase enzymes). Because our method is based on cDNA, if a given pair of primers is able to amplify a gene using a DNA template, then it is more reliable to use this pair to prime the synthesis of the complementary strand of the target cDNA compared to the retro-transcription of RNA.

Our method is less expensive in comparison to the commercial kits or reagents required for RT-PCR amplification and for Northern blot hybridisation and also reduces the risks associated with the use of toxic reagents involved in the detection procedures.

During recent years, RNA-dependent DNA polymerase enzymes were constantly modified, and some of them now can also function at a high temperature, such as 50°C, increasing the stringency and the specificity of those reactions using GSPs [24]. Yet, after a short step of annealing the primer, our method is performed at an even higher temperature, at 72°C, allowing more specificity when compared to RT-PCR.

**Figure 5:**
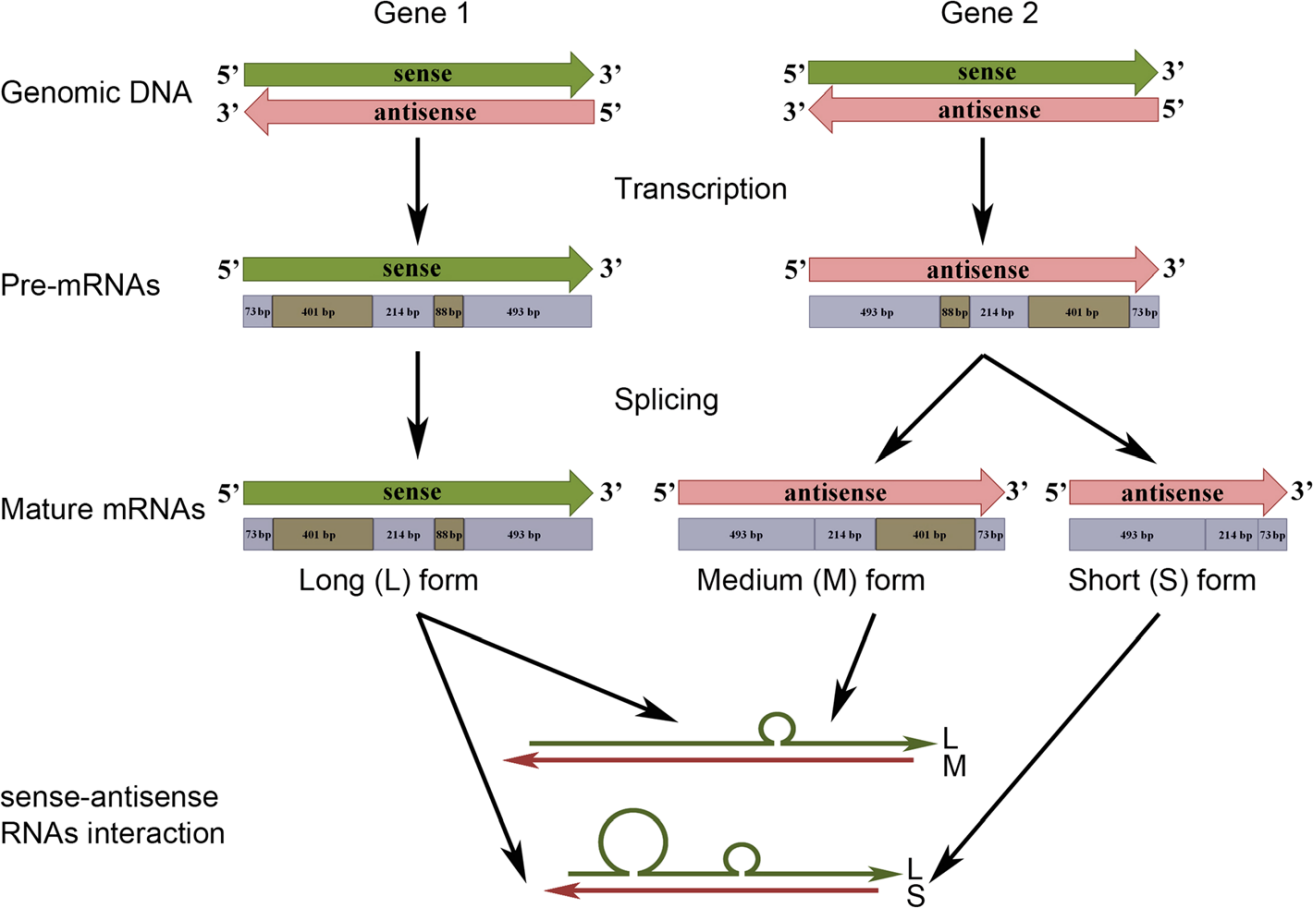
**Summary of the predicted pathway in olive anthers. **The sense (green) and antisense (pink) transcripts are coded by genes located at two distinct loci. The sense transcripts are transcribed from locus 1, and the pre-mRNA is not spliced, whereas the antisense transcripts are transcribed from locus 2, and the pre-mRNA can undergo two different splicing events to produce a molecule lacking the small intron or both introns. These splicing variant events are possible because the nucleotide sequences the introns of the antisense transcripts are flanked by the splice donor (GU) and acceptor (AG) sites, which are not present in the sense transcript nucleotide sequences. The interaction between the sense and antisense transcripts can result in two different secondary structures, consisting of one single bulge, when only the small intron is excised, and two bulges, when both introns are excised. The introns are indicated in light brown and the exons in light blue.

In addition to refer a new method to detect NATs within a specific tissue or organ, to the best of our knowledge, this study is the first to report a NAT occurring in olive and the *Oleaceae* family. We demonstrated that antisense transcripts of *OeSLG* are specifically expressed in olive anthers and that this orientation allowed the excision of two introns. We further hypothesised that this antisense transcript belongs to the *trans*-NAT class and is expressed from a locus that is separate from the sense protein-coding transcript due to the SNPs found between the sense and antisense nucleotide sequences, a feature that is not possible in the case of *cis*-NATs. As the antisense transcripts were found to be specifically expressed in anthers, we also hypothesised that alternative transcriptional regulatory elements are involved. Furthermore, according to the sense and antisense nucleotide sequences and taking into account the possible interaction between the two forms (see Figure 3), we hypothesised that interactions between sense and antisense *OeSLG* transcripts occur along the entire length of the sequences, producing one or two bulges due to the splicing of one or two introns from the medium and short forms, respectively (Figure 5). Four main mechanisms are proposed for the antisense-mediated regulation of sense mRNA: mechanisms related to transcription, RNA-DNA interactions, RNA-RNA interactions in the nucleus and RNA-RNA interactions in the cytoplasm [27]. Because we found mature poly-adenylated transcripts of both the sense and antisense orientations and only the sense transcript is predicted to encode for an open reading frame, corresponding to the expected *OeSLG* protein, we speculated that this NAT of olive belongs to the RNA-RNA-interactions in the cytoplasm category. According to the theory, the cytoplasmic sense-antisense RNA duplex can affect the mRNA stability or translation, may cover miRNA-binding sites or serve as a hairpin template for generate endogenous siRNAs [27]. Nevertheless, further studies should be aimed at elucidating the biological function of these NATs in olive anthers. 

## Conclusions

To the best of our knowledge, this method is the first that combines the more amenable condition of using DNA instead of RNA with the polymerisation and cleavage activity of DNA polymerase and exonuclease enzymes, respectively, allowing for the precise detection of NATs in living organisms and providing several advantages compared to the conventional methods currently used. We also characterized the first NATs in *Oleaceae* family and we demonstrated its expression restricted to the anthers. Further studies should be aimed at elucidating the biological function of these NATs in olive anthers. Furthermore, since NATs are found in multiple eukaryotes, our method can be easily applied to a wide range of organisms, including human, mice and yeast.

## Methods

### Plant material, RNA extraction and cDNA synthesis

Total RNA was obtained from the pistils, anthers, leaves, branches and roots of the olive cultivar Leccino using the RNeasy Plant Mini Kit (Qiagen) and was treated by DNase (Qiagen) according to the manufacturer’s protocols. The RNA samples were analysed by agarose gel electrophoresis to test the integrity and were quantified using a nano-drop spectrophotometer.

### Method

The method consists of four main steps: first-strand cDNA synthesis by RNA retro-transcription, synthesis of the complementary strand of target cDNAs, degradation of the ssDNAs in solution, amplification by PCR of those dsDNAs that were not degraded (Figure 1).

First-strand cDNAs were produced from 250 ng of total RNA by RT-PCR and SuperScript III (Invitrogen) using Oligo(dT) primers. The samples were then treated with RNaseH (Invitrogen) according to the manufacturer’s protocol.

The synthesis of the complementary strand of the target cDNA was performed using 5 μl of dilute cDNA (1:25) as the template in a final volume of 25 μl of a normal PCR reaction (2.5 μl 10 X Reaction Buffer, 1.2 50 mM MgCl_2_, 1 μl 10 mM dNTPs, 1 U Taq and 13.1 μl MilliQ water). Then, 1 μl of 10 μM 5’-tagged primer based on the strand to be amplified was added: a forward primer to detect the sense transcript and a reverse primer to detect the putative antisense transcripts, as the cDNA is a ssDNA orientated in the 3’→5’ direction of the mRNA (5’→3’). Along with the gene-specific primer, 1 μl of 10 μM 5’-tagged forward primer annealing to Elongation Factor α1, a housekeeping gene, was added to each reaction for a positive control. An initial denaturation step at 94°C for 5 minutes, subsequent annealing at 60°C (the melting temperature of the primer) for 30 seconds and an elongation phase at 72°C for 30 minutes allowed a conventional DNA-dependent DNA polymerase to synthesise the complementary strand of the target cDNA. At the end of this step, those cDNAs having homology with the primer will be dsDNA, whereas all of the other cDNAs will remain as ssDNA.

The degradation of the ssDNA was performed by a treatment with exonuclease, an enzyme that recognises ssDNAs, releasing deoxyribonucleoside 5’-monophosphates in a stepwise manner. Exonuclease I (2 U, Fermentas) was added to each reaction and incubated at 37°C for 1 hour, followed by 15 minutes at 80°C to inactivate the enzyme. This step degraded all of the ssDNAs; in other words, those cDNAs that are not preserved by the complementary strand formed during the previous step will be degraded.

To amplify the transcripts that are preserved from the exonuclease activity by the complementary strand, a PCR-amplification using 5 μl of an ExoI-treated reaction was performed using the TAG as the forward primer in combination with the reverse gene-specific primer to detect the sense transcripts and to the forward gene-specific primer to detect the putative antisense transcripts (Table 1).

**Table 1:** Name and sequence (5’ – 3’) of primers used for the experiments

**Primer name**	**Primer sequence**
OeSLG_full-length_FOR	ATGGAGAAATCGATTAAAGATATA
OeSLG_full-length_REV	CTAAGAAGAAGCCATTCTAATGTAAATA
OeSLG_full-length_5'-tag_FOR	GGCAGTATCGTGAATTCGATGCATGGAGAAATCGATTAAAGATATA
OeSLG_full-length_5'-tag_REV	GGCAGTATCGTGAATTCGATGCCTAAGAAGAAGCCATTCTAATGTAAATA
OeSLG_internal_FOR	ATTACCAGACGCACAATATTCCACG
OeSLG_internal_REV	TGCAGCATTCCAACCGAAGTTC
OeEF_FOR	TGCACAGTTATTGATGCTCCA
OeEF_REV	GGGCTCCTGAATCTGGTCAA
OeEF_5'-tag_FOR	GGCAGTATCGTGAATTCGATGCTGCACAGTTATTGATGCTCCA
primer_TAG	GGCAGTATCGTGAATTCGATGC

One reaction was also performed without primers to test for possible cDNA secondary structures, triggering the primer-independent synthesis of complementary strands. This reaction was also used to test whether the method can be applied to detect possible interactions between the sense and antisense transcripts.

### Test of interaction between sense and antisense OeSLG

To test the interaction between the sense and antisense *OeSLG*, the full-length sequences were cloned and amplified using primers designed to include the start and stop codons. The products were extracted with phenol-chloroform-isoamyl alcohol and precipitated with ethanol, separated using agarose gel electrophoresis to evaluate the integrity and quantified using a nano-drop spectrophotometer. To employ a stoichiometric balance between the antisense (780 bp) and the sense (1,269 bp) transcripts, we used a 1:1.62 ratio of the two transcripts. Three different reactions were performed. The first included 1,620 ng of sense *OeSLG*, the second consisted of 810 ng and 500 ng of sense and antisense *OeSLG*, respectively, and the third consisted of 1,000 ng of antisense *OeSLG*. Annealing buffer (10 mM Tris [pH 8,0], 50 mM NaCl, and 1 mM EDTA [pH 8,0]) was added to the DNA to a final volume of 50 μl, and the reactions were heated at 94°C for five minutes and cooled at room temperature for three hours. An aliquot was assayed by 1% agarose gel electrophoresis.

### RT-PCR analysis using gene-specific primers

In order to get a further validation of our method, we performed RT-PCRs using tagged gene-specific primers (GSPs) in RNAs isolated from pistils and anthers. In particular, we used a reverse primer (*i*.*e*. OeSLG_full-length_5’-tag_REV) designed to specifically detect the sense transcripts and a forward primer (*i*.*e*. OeSLG_full-length_5’-tag_FOR) designed to specifically detect the antisense transcripts. Furthermore we added to each reaction OeEF_REV to detect the *Elengation Factor α1* used as housekeeping gene. The subsequent amplifications were carried out using primer_TAG in combination with OeSLG_internal_FOR to detect sense transcripts and in combination with OeSLG_internal_REV to detect antisense transcripts. *OeEF* was amplified by using OeEF_FOR and OeEF_REV (Table 1).

## Competing interests

The authors declare that they have no competing interests.

## Authors’ contributions

The experiments were conceived and performed by SC. The manuscript was written by SC and GB. GB is the scientific responsible person of the research grant. Both authors read and approved the final manuscript.

## Supplementary Material

Additional file 1 **Nucleotide sequences of the *OeSLG *sense transcript and the *OeSLG *medium and short antisense transcripts.** Click here for file
