# Supplementary material for: Development of a rapid and inexpensive method to reveal natural antisense transcripts
Source: Plant Methods. 2012 Sep 12;8:37. doi: 10.1186/1746-4811-8-37 (PMC3490877; doi:10.1186/1746-4811-8-37)
Supplement: Additional file 1 — Nucleotide sequences of the OeSLG sense transcript and the OeSLG medium and short antisense transcripts. [file 1746-4811-8-37-S1.docx]

**Additional file 1**

>OeSLG_long_form_sense

ATGGAGAAATCGATTAAAGATATATCCCTTCTTCTTCTCTTAACATCCATCTTGTCCATCCTAGAGATTTCACCTGCAATTGATACCATTAGCACAACTCAGAGCCTCAAAGATGGAGATACCATGGTTTCATCAGGGGGAACCTTCGAACTGGGATTTTTCAGCCCAGGTGACTCCAAGAATCGGTACGTGGGAATTTGGTATAAGAAGGTGCCTAGCATAACAGCAGTTTGGGTCCTCAACAGAGAAATTCCGTTAAATAGTAGATCAGGCATACTAAAATTCAATGAGCTAGGCCACTTGGTTCTTGTGAATGACACTAATAACCTCTTGTGGTCTTCAAATACATCAAGAATTGCAGGAACTCCAATTTTGCAATTGCTGGACTCGGGAAATCTTGTTCTTCGAGAAGCAAATGATGATAATCCGGAAAATTTCCTTTGGGAGAGTTTCGATTATTTAAGTGACACTTACCTACCAGGCATGAACTTCGGTTGGAATGCTGCAACAGGTGTACAGAACTATTTGTCATCATGGACGAGCAATGAGGATCCGGCTCCAGGAGATCTTACATTTTACCTGGATCCAACTGGATATCCACAAGTCTTCATCAAAAGAGGCACAGGTGCCATATACAGAATGGGACCTTGGAATGGTCTTCGCTTTAGTGGAACACCATATGTGAGTCCTACATTCAGACATGGAATATTCAAGAATAAGAACACGACGTACTATAGAGAAGACTCCAATGACAAATCTGTTATTTCAAGGGTTACCCTGAATCAGAGTGGTGTTGTACAGCGCTGGGTATGGGTCGATCGAACTAGAGGTTGGGTCCTCTACTTGACTGTACCAAAAGATGATTGTGACACTTATAGTGGGTGTGGAGCTTATGGGACTTGCTATATTGGGAATTCTCCGGCTTGTGGATGTCTGAGTAAATTTCAGCCAAAAGATCCAGAAGGATGGAATAAGGGAGATTGGTCAAATGGGTGCATTAGAAGGACTCCCTTAAATTGTCAAGAAGGTGATGTATTTTTGAAGTATTCTAGCGTTAAATTACCTGACGCACAATATTCCACGTATAATGAGAGTATGACACTTGACGAATGCGAAGTGAAGTGTTTACAGAATTGTTCTTGTATGGCATATTCACAATTGGATATCAGGCGAGGGAGTGGATGCCTGTTTTGGTTTCGAGAATTGATTGACATCAGAGATATGTCTTCGGACGGACAAGATATTTACATTAGAATGGCTTCTTCTTAG

>OeSLG_medium_form_antisense

CTAAGAAGAAGCCATTCTAATGTAAATATCTTGTCCGTCCGAAGACATATCTCTGATGTCAATCAATTCTCGAAACCAAAACAGGCATCCACTCCCTCGCCTGATATCCAATTGTGAATATGCCATACAAGAACAATTCTGTAAACACTTCACTTCGCATTCGTCAAGTGTCATACTCTCATTATACGTGGAATATTGTGCGTCAGGTAATTTAACGCTAGAATACTTCAAAAATACATCACCTTCTTGACAATTTAAGGGAGTCCTTCTAATGCACCCATTTGACCAATCTCCCTTATTCCATCCTTCTGGATCTTTTGGCTGAAATTTACTCAGACATCCACAAGCCGGAGAATTCCCAATATAGCAAGTCCCATAAGCTCCACACCCACTATAAGTGTCACAATCATCTTTTGGTACAGTCAAGTAGAGGACCCAACCTCTAGTTCGATCGACCCATACCCAGCGCTGTACAACACCACTCTGATTCAGGGACTCACATATGGTGTTCCACTTAAGCGAAGACCATTCCAAGGTCCCATTCTGTATATGGCACCTGTGCCTCTTTTGATGAAGACTTGTGGATATCCAGTTGGATCCAGGTAAAATGTAAGATCTCCTGGAGCCGGATCCTCATTGCTCGTCCATGATGACAAATAGTTCTGTACACCTGTTGCAGCATTCCAACCGAAGTTCATGCCTGGTAGGTAAGTGTCACTTAAATAATCGAAACTCTCCCAAAGGAAATTTTCCGGATTATCATCATTTGCTTCTCGAAGAACAAGATTTCCCGAGTCCAGCAATTGCAAAATTGGAGTTCCTGCAATTCTTGATGTATTTGAGGACCACAAGAGGTTATTAGTGTCATTCACAAGAACCAAGTGGCCTAGCTCATTGAATTTTAGTATGCCTGATCTACTATTTAACGGAATTTCTCTGTTGAGGACCCAAACTGCTGTTATGCTAGGCACCTTCTTATACCGAATTCCCACGTACCGATTCTTGGAGTCACCTGGGCTGAAAAATCCCAGTTCGAAGGTTCCCCCTGATGAAACCATGGTATCTCCATCTTTGAGGCTCTGAGTTGTGCTAATGGTATCAATTGCAGGTGAAATCTCTAGGATGGACAAGATGGATGTTAAGAGAAGAAGAAGGGATATATCTTTAATCGATTTCTCCAT

>OeSLG_short_form_antisense

CTAAGAAGAAGCCATTCTAATGTAAATATCTTGTCCGTCCGAAGACATATCTCTGATGTCAATCAATTCTCGAAACCAAAACAGGCATCCACTCCCTCGCCTGATATCCAATTGTGAATATGCCATACAAGAACAATTCTGTAAACACTTCACTTCGCATTCGTCAAGTGTCATACTCTCATTATACGTGGAATATTGTGCGTCAGGTAATTTAACGCTAGAATACTTCAAAAATACATCACCTTCTTGACAATTTAAGGGAGTCCTTCTAATGCACCCATTTGACCAATCTCCCTTATTCCATCCTTCTGGATCTTTTGGCTGAAATTTACTCAGACATCCACAAGCCGGAGAATTCCCAATATAGCAAGTCCCATAAGCTCCACACCCACTATAAGTGTCACAATCATCTTTTGGTACAGTCAAGTAGAGGACCCAACCTCTAGTTCGATCGACCCATACCCAGCGCTGTACAACACCACTCTGATTCAGGGACTCACATATGGTGTTCCACTAAAGCGAAGACCATTCCAAGGTCCCATTCTGTATATGGCACCTGTGCCTCTTTTGATGAAGACTTGTGGATATCCAGTTGGATCCAGGTAAAATGTAAGATCTCCTGGAGCCGGATCCTCATTGCTCGTCCATGATGACAAATAGTTCTGTACACCTGTTGCAGCATTCCAACCGAAGTTCATGCCTGGTAGGTGAAATCTCTAGGATGGACAAGATGGATGTTAAGAGAAGAAGAAGGGATATATCTTTAATCGATTTCTCCAT
